# Self-tracking via smartphone app: Potential tool for athletes’ recovery self-management? A survey on technology usage and sleep behaviour

**DOI:** 10.1007/s12662-022-00812-3

**Published:** 2022-04-29

**Authors:** Sarah Jakowski

**Affiliations:** Unit of Sport Psychology, Faculty of Sport Science, Ruhr University Bochum, Gesundheitscampus Nord 10, 44801 Bochum, Germany

**Keywords:** Monitoring, Sleep-wake pattern, Self-regulation, User behavior, Self-quantification

## Abstract

Self-tracking technologies are possible approaches to support recovery self-management activities for athletes. These may have become even more appealing due to stay-at-home restrictions as part of the 2020 pandemic regulations. This study examined user behaviour of smartphone and wearable technologies among 217 athletes (29% women, *M* age = 26.9 ± 7 years). The online survey comprised demographic questions and standardised questionnaires to assess usage of technologies, sleep quality (Pittsburgh Sleep Quality Index), daytime sleepiness (Epworth Sleepiness Scale), attitudes about sleep (Dysfunctional Beliefs and Attitudes about Sleep Scale), bedtime procrastination (Bedtime Procrastination Scale), and self-control (Brief Self-Control Scale). Fitness apps (46.5%) were more popular than sleep apps (15.7%) followed by nutrition apps (12%). The correlation between sleep apps and the other two apps indicate that non-users of sleep apps are probably also non-users of fitness or nutrition apps. Wearables were more frequently used to track fitness activities (36.9%) than sleep (17.5%). Considering sex, type of sport, competition participation, and training volume, no remarkable characteristics among users versus non-users of sleep apps were identified. There were also no significant differences among sleep indices between sleep app users and non-users. However, self-control was highest among sleep app users compared to non-users (*d* = 0.58). Despite 34.1% being identified as *poor sleepers*, behavioural sleeping patterns were within normal range. The results imply that athletes are not as attracted to self-tracking technologies as expected, which makes them less vulnerable to unsubstantiated feedback and inappropriate interventions by those tools. This serves as a starting point to explore the potential of self-tracking ambulatory assessment for physical activity and sleep behaviour of athletes in the post-pandemic era.

## Introduction

Performance and recovery management increasingly comprises monitoring external and internal training load and the athlete’s response. Monitoring may help to maintain performance capacity and health, including injury prevention and optimizing recovery. From a holistic point of view, the main goal is to achieve balance between effective training impulses and sufficient recovery and rest periods (Kellmann et al., 2018). Monitoring systems have evolved over the past years and a number of approaches are available to athletes, coaches, and sport scientists. While training-related assessments need to be tailored to the specific characteristics of each type of sport, global approaches such as subjective self-report measures of well-being and recovery-stress states are available on a broad level (Kölling & Kellmann, 2020).

However, monitoring is only half the battle. Recovery self-management and self-regulation have to be considered as an important task of successful athletes—on the elite as well as on the leisure level. Recognising one’s current recovery need and acting accordingly, i.e. initiating and applying appropriate measures, is crucial (Balk & Englert, 2020). Since sleep is considered one of the most efficient and important recovery strategies, it seems an obvious target when it comes to optimizing regeneration (Kölling, Duffield, Erlacher, Venter, & Halson, 2019; Walsh et al., 2021). At the same time, sleep is highly vulnerable to external and internal stressors, which are prevalent among athletic as well as university populations (Doherty, Madigan, Nevill, Warrington, & Ellis, 2021; Wang & Bíró, 2021). While training/competition and schedule-related stress may be one reason for unrestful or insufficient sleep, dysfunctional cognitions and behaviours may be another influencing factor (Hiller, Johnston, Dohnt, Lovato, & Gradisar, 2015; Kölling et al., 2019; Kroese, Evers, Adriaanse, & de Ridder, 2016).

As the sleeping process is inherently beyond the sleeper’s consciousness, objective assessments that provide information about sleep-related events may be quite appealing. While standardised measures such as polysomnography and actigraphy are the method of choice in research and sleep medicine, these may be too costly and complex for the average athlete (Halson, 2019; Shelgikar, Anderson, & Stephens, 2016). Self-tracking wearable technologies and smartphone applications have made the approach to one’s sleep behaviour more easily accessible, although their validity and reliability are considered highly doubtful (Khosla et al., 2018). Possible benefits are that they enhance the user’s awareness of their sleeping patterns (Watson, Lawlor, & Raymann, 2019). However, several drawbacks are discussed among experts and even negative effects of the usage of smartphone applications may be observed (Baron, Abbott, Jao, Manalo, & Mullen, 2017; Shelgikar et al., 2016; Van den Bulck, 2015). For instance, users may become too obsessed with self-optimisation and overemphasise the dubious feedback. Especially people with subjective sleep complaints and those concerned about their health are assumed to be prone to misguidance via self-tracking consumer technologies (Baron et al., 2017). It is hypothesised that ambitious and performance-oriented athletes represent a target group that is attracted by those technologies. This assumption needs yet to be confirmed.

Moreover, the 2020 confinements due to the coronavirus disease 2019 (COVID-19) significantly affected sleep and mental health of Australian athletes (Facer-Childs, Hoffman, Tran, Drummond, & Rajaratnam, 2021) as well as the psychobiosocial state of Italian athletes (di Fronso et al., 2022). While physical activity of healthy adults generally declined during lockdown in the USA, this decline was buffered by the use of fitness apps (Yang & Koenigstorfer, 2020). It is possible that the lockdown regulations in Germany raised athletes’ self-awareness and openness to explore their physical activity and sleep. The restricted access to sport facilities and organised activities as well as the promoted social distancing and staying-at-home measures may have contributed to sensitise the individuals to focus more on their psychophysiological state. As training and exercise opportunities were limited, more attention could be paid to assess and optimise recovery processes. In this context, athletes may consider inexpensive and easy-to-use tools as a convenient opportunity to track and analyse physical activity, sleep, and nutrition. Therefore, it is hypothesised that the majority of athletes will report to use either smartphone applications or wearable technologies to support their training and recovery management.

In summary, athletes on either performance level in the pursuit of optimising performance and recovery constitute a vulnerable group that might be attracted to self-tracking technologies. If usage was highly prevalent, the characteristics of users need to be investigated. For instance, it is conceivable that users either have poor sleeping patterns and rely on the self-tracking technologies to deal with these issues or, on the contrary, that users show better sleeping patterns than non-users because they are able to manage the necessary requirements with the help of these technologies. The former scenario would lead to increased concerns of researchers and practitioners, as the above-mentioned negative effects need to be addressed. If athletes place too much confidence into customer technologies, they may become less accessible for more scientific approaches. The latter scenario, on the other hand, would indicate the potential of using self-tracking technologies to increase athletes’ self-awareness and responsibility. There is currently a lack of data on user behaviour and current sleeping patterns. Therefore, this manuscript evaluates the experience of German athletes with self-tracking technologies, especially smartphone apps and wearable technologies. The second aim of this study was to analyse the young athletes’ behavioural sleeping patterns and how these differ among users and non-users of sleep self-trackers. Furthermore, correlations of sleep parameters with participants’ characteristics will be examined to shed more light on the state of athletes in a period of time that was characterised by gradual reversed restrictions of exercise and training opportunities based on coronavirus disease 2019 (COVID-19) pandemic regulations in Germany.

## Procedure

Data were collected between May and July 2020 via online surveys which were distributed via personal contacts and social media channels. After receiving information about the purpose of the study and reading the confidentiality statement of data processing, respondents provided informed consent by ticking a specific box to start participation. The survey consisted of a demographic questionnaire and standardised questionnaires to assess usage of technologies, sleep quality, daytime sleepiness, attitudes about sleep, bedtime procrastination and self-control. Ethical clearance was secured prior to the study from the local ethical committee (EKS-V-10/2020). On average, it took participants 14.7 ± 4.7 min to complete the survey.

## Participants

The sample consisted of 217 participants, with 29% (*n* = 63) female and 71% (*n* = 154) male respondents. Mean age was 26.9 ± 7 years and average training volume was 10.7 ± 5.4 h per week. The majority declared regular competition participation (*n* = 150; 69%), with 22.7% (*n* = 49) competing on the national or international level. The majority (58.5%) participated in individual sports and 41.4% were assigned to team sports. Only 10% (*n* = 22) stated to be in the regular season at the time of answering the survey, while 22% (*n* = 48) were in the preseason and 27% (*n* = 59) in the off-season phase. Only 5 participants were professional athletes, while just over half (51.6%) reported to be employed and 40.6% were school or university students.

## Instruments

### Stage model of self-tracking technology use

The use of smartphone apps and wearables was assessed following the stage model approach described by König, Sproesser, Schupp, and Renner (2018). The participant’s stage in the adoption process of apps that track (a) sleep (*sleep apps*), (b) physical activity (*fitness apps*), and (c) nutrition (*nutrition apps*) as well as wearables that track (d) sleep and (e) physical activity was assessed via five statements which depict each of the different stages. Respondents were then categorised as being *unengaged* (stage 1; “I have never thought about using an app/wearable for that”), *decided to act* (stage 2; “I have thought about using an app/wearable for that, but so far I did not do it”), *decided not to act* (stage 3; “I have thought about using an app/wearable for that, but it is not necessary for me to do it”), *acting* (stage 4; “I am currently using an app/wearable for that and intend to continue to use it”), and being *disengaged* (stage 5; “I have used an app/wearable for that, but I do not use it anymore”). Thus, only stage 4 includes current users, while the other stages encompass non-users.

### Sleep quality

Sleep quality was assessed with the Pittsburgh Sleep Quality Index (PSQI) which encompasses 19 self-rating items regarding the previous 4 weeks (Buysse, Reynolds III, Monk, Berman, & Kupfer, 1989). The items are summarised to seven component scores, ranging from 0 to 3 each, which reflect the following sleep-related domains: subjective sleep quality, sleep latency, sleep duration, sleep efficiency, sleep disturbances, use of sleep medication, and daytime dysfunction. The global PSQI score is calculated by the sum of these domains and ranges from 0 to 21. The general cut-off to differ between *good* and *poor* sleep quality is considered as PSQI ≥ 6, while values > 10 reflect *excessive sleep problems* (Buysse et al., 1989). Psychometric properties of the German version support its suitability for measuring sleep quality (Hinz et al., 2017). In the current study, Cronbach’s alpha for the total score was α = 0.70.

### Daytime sleepiness

Daytime sleepiness was assessed via the 8‑item Epworth Sleepiness Scale (ESS) which captures the proneness of falling asleep in different everyday situations (Johns, 1991). Each statement is assessed on a rating scale ranging from 0 (*would never doze*) to 3 (*high chance of dozing*). Values are then summed up to calculate the ESS score that ranges from 0 to 24, with ESS scores ≤ 10 considered as *normal* and ESS ≥ 16 as *severe* daytime sleepiness (Johns, 1991). Good validity and reliability of the German version was reported by Bloch, Schoch, Zhang, and Russi (1999). Reliability of the scale was acceptable in the present study with α = 0.74.

### Sleep-related beliefs and attitudes

The 16-item version of the Dysfunctional Beliefs and Attitudes about Sleep Scale (DBAS) was used to identify sleep-related cognitions (Morin, Vallières, & Ivers, 2007). Each statement that contains beliefs, attitudes, expectations, appraisals, or attributions is rated on a scale ranging from 0 (*strongly disagree*) to 10 (*strongly agree*), where higher values indicate more dysfunctional cognitions. Accordingly, the sum score of the DBAS ranges from 0 to 160. The German version showed good psychometric properties (Weingartz & Pillmann, 2009) and the reliability coefficient revealed good homogeneity in the present sample with α = 0.84.

### Bedtime procrastination

As a determinant of healthy sleeping behaviour, the Bedtime Procrastination Scale (BPS) was used to assess the participants’ probability to delay going to bed without external reasons for doing so (Kroese, de Ridder, Evers, & Adriaanse, 2014). The BPS contains nine items that are rated on a scale ranging from 1 (*never*) to 5 (*always*) which are summarised to a global mean score. Higher scores indicate higher bedtime procrastination. According to Kroese et al. (2016), bedtime procrastination is a strong predictor of experiencing insufficient sleep. Moreover, the relation between self-regulation and insufficient sleep was mediated by bedtime procrastination. Internal consistency of the German BPS was found to be high in the present investigation (α = 0.92).

### Self-control

The Brief Self-Control Scale (BSCS) was used as an indicator of self-regulation ability (Tangney, Baumeister, & Boone, 2004). The 13 items include statements related to self-control failure covering control over thoughts, emotional control, impulse control, performance regulation, and habit breaking. Each statement (e.g. “I am good at resisting temptations”) is rated on a scale from 1 (*not at all like me*) to 5 (*very much like me*) and then summed up, with higher scores indicating higher degrees of self-control. Evidence for the reliability and validity of the German version was reported by Bertrams and Dickhäuser (2009) and Sproesser, Strohbach, Schupp, and Renner (2011), which is also supported in the present sample with α = 0.84. Moreover, Kroese et al. (2016) highlighted the role of self-control for sleep-related indicators such as experienced insufficient sleep and discrepancy between intended and actual bedtimes.

## Statistical analyses

Data processing and analysis was performed with the software SPSS 26 (IBM, Armonk, NY, USA). To analyse the prevalence of the use of self-tracking smartphone apps and wearables, descriptive statistics will be presented in absolute and relative numbers according to the stage model described above (König et al., 2018). Chi-square tests were performed to analyse the relationship between the five stages regarding sleep apps and (a) sex, (b) regular competition participation, (c) type of sport, (d) fitness app use, (e) nutrition app use, (f) use of wearable sleep trackers, and (g) use of wearable fitness trackers. In case of significant results (*p* ≤ 0.05), Cramer’s *V* will be reported to indicate the size of correlation. To address the second aim of the study, differences between the sleep app use stages and sleep-related variables were analysed via one-way analyses of variance (ANOVA) with Bonferroni corrected post hoc tests in case of significant main effects (*p* ≤ 0.05). Levene tests were conducted to test for homogeneity of variances and this precondition was fulfilled by each variable. In particular, the habitual time spent in bed, sleep duration, and sleep efficiency (expressed as the percentage of sleep duration relative to time in bed) as well as sleep quality (PSQI), daytime sleepiness (ESS), sleep-related cognitions (DBAS), bedtime procrastination (BPS), and self-control (BSCS) were examined. Finally, correlations among the sleep-related constructs as well as with the participants’ age and weekly training volume were investigated by Pearson’s product–moment correlation coefficients.

## Results

### Usage of self-tracking technologies

**Fig. 1:**
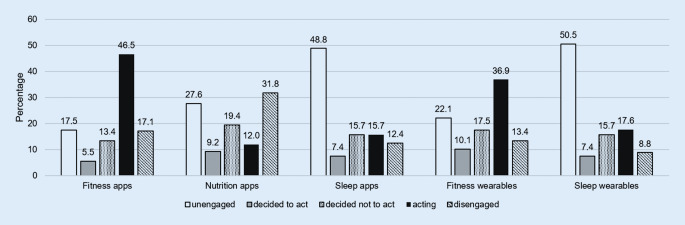
Distribution of the user behaviour of smartphone apps and wearables

Figure 1 shows the distribution of each stage of the adoption process of the different technologies. Evaluating the stages of the adoption process of sleep apps, almost half of the respondents (*n* = 106) were categorised as *unengaged* (stage 1) and *n* = 27 as *disengaged* (stage 5), while merely *n* = 34 turned out to be *acting* (stage 4). The smallest proportion of respondents was found among the category *decided to act* (stage 2; *n* = 16). Duration of sleep app usage was less than 1 year for most of the respondents (*n* = 23), followed by 1–2 years (*n* = 19) and only 5 participants reported to track sleep via apps for more than 2 years. About one third of the respondents (*n* = 79; 36.4%) tended to agree that sleep tracking apps are useful or were undecided (*n* = 84; 38.7%), while 25% (*n* = 54) tended to disagree with this statement.

Table 1 presents the results of stage distributions for the different categories that were analysed. More than one third of the female athletes (38.1%) responded to be *unengaged* and 19% were categorised to be *disengaged* and *acting*, respectively. Among the male athletes, over the half (53.2%) were categorised as *unengaged*, while 9.7% were *disengaged* and 14.3% *acting*. There was no significant correlation among the distribution of the stages and sex (χ^2^ = 5.9, *df* = 4, *p* = 0.207).

**Table 1:** Distribution of the frequencies (*n*) for the 5 stages of the adoption process of sleep apps

	Stage 1 (unengaged)	Stage 2 (decided to act)	Stage 3 (decided not to act)	Stage 4 (acting)	Stage 5 (disengaged)	Total
*Total*	106	16	34	34	27	217
*Women*	24	5	10	12	12	63
*Men*	82	11	24	22	15	154
*Competition participation*						
Yes	72	10	23	30	15	150
No	34	6	11	4	12	67
*Individual sports*	58	8	24	21	16	127
*Team sports*	48	8	10	13	11	90
*Fitness app use*						
Unengaged	31	1	4	2	0	38
Decided to act	7	3	1	1	0	12
Decided not to act	9	3	13	2	2	29
Acting	38	9	12	27	15	101
Disengaged	21	0	4	2	10	37
*Nutrition app use*						
Unengaged	43	2	8	5	2	60
Decided to act	8	4	1	4	3	20
Decided not to act	22	2	11	3	4	42
Acting	4	5	2	9	6	26
Disengaged	29	3	12	13	12	69
*Sleep wearable use*						
Unengaged	89	2	9	3	6	109
Decided to act	1	10	1	4	0	16
Decided not to act	7	2	20	1	4	34
Acting	6	2	0	26	4	38
Disengaged	3	0	3	0	13	19
*Fitness wearable use*						
Unengaged	38	0	4	2	4	48
Decided to act	9	6	2	4	1	22
Decided not to act	19	2	13	2	2	38
Acting	30	8	5	26	11	80
Disengaged	10	0	10	0	9	29

Of the 150 competition-oriented athletes, 48% were categorised as *unengaged* and 10% as *disengaged*, while 20% reported to be *acting* (Table 1). The distribution of the stages did not correlate significantly between competitive and noncompetitive athletes (χ^2^ = 8.58, *df* = 4, *p* = 0.072). Comparing the type of sport, 45.7% of individual athletes and 53.3% of team athletes were *unengaged*, 12.6 and 12.2% were *disengaged* individual and team athletes, respectively (Table 1). 16.5% of the individual athletes and 14.4% of the team athletes were categorised as *acting*. There was no significant correlation among the distribution of the stages compared by type of sport (χ^2^ = 3.3, *df* = 4, *p* = 0.508). Comparing the average training volume between the categories, no significant difference was found either (*F* (4, 211) = 1.57, *p* = 0.182).

Regarding the use of fitness apps, 46.5% (*n* = 101) reported to be *acting* (Fig. 1), with a proportion of 26.7% who were at the same time *acting* and 37.6% who were *unengaged* among sleep apps (Table 1). The use of fitness apps correlated significantly with the use of sleep apps (χ^2^ = 69.98, *df* = 16, *p* < 0.001), whereas the effect was small (Cramer’s *V* = 0.28, *p* < 0.001).

In terms of the use of nutrition apps, 12% of the respondents reported to be *acting *(Fig. 1), with a proportion of 34.6% who were at the same time *acting* and 15.4% who were *unengaged* regarding sleep apps, respectively (Table 1). There was a significant, but small correlation between the use of nutrition apps and sleep apps (χ^2^ = 49.88, *df* = 16, *p* < 0.001, Cramer’s *V* = 0.24, *p* < 0.001).

Considering the application of wearable technologies to track sleep, 17.6% of the respondents were categorised as *acting* (Fig. 1), with a proportion of 68.4% who were at the same time *acting* and 15.8% who were *disengaged* regarding the use of sleep apps, respectively (Table 1). Half of the participants (50.5%) reported to be *unengaged* in terms of sleep wearables, while 8.8% were *disengaged *(Fig. 1). There was a statistically significant, strong correlation between the use of sleep wearables and sleep apps (χ^2^ = 312.31, *df* = 16, *p* < 0.001, Cramer’s *V* = 0.60, *p* < 0.001). The majority reported to use sleep tracking wearables less than 1 year (*n* = 25) and not more than 2 years (*n* = 10), whereas 10 participants used them for more than 2 years. About one third (35%) tended to agree or were undecided (41.5%) that sleep tracking wearables are useful, whereas 23.5% tended to disagree with this statement.

More than one third (36.9%) of the participants were categorised as *acting* among the use of wearable fitness trackers (Fig. 1), with a proportion of 32.5 and 37.5% who were simultaneously categorised as *acting* and *unengaged* regarding sleep apps, respectively (Table 1). There was a significant, moderate correlation between the use of fitness wearables and sleep apps (χ^2^ = 90.08, *df* = 16, *p* < 0.001, Cramer’s *V* = 0.32, *p* < 0.001).

### Sleeping patterns

On average, participants reported a habitual time in bed of 8.2 h ± 66.6 min and subjective sleep duration of 7.8 h ± 65.8 min, resulting in a sleep efficiency of 95.2 ± 3.7%. The overall PSQI score was 5.3 ± 2.9, with 59% (*n* = 128) categorised as having *good *sleep quality, 34.1% (*n* = 74) as having *poor *sleep quality and 6.5% (*n* = 14) revealing *chronically disturbed sleep*. Sleep quality scores did not differ significantly between the stages of the adoption process of sleep apps (*F* (4, 211) = 37.93, *p* = 0.353). However, on the descriptive level, those participants categorised as *decided not to act* presented the lowest score (*M* = 4.4 ± 2.1), while average scores among all the other groups were above 5, but below the cut-off (PSQI ≥ 6). The highest average score was prevalent in the category *disengaged* (*M* = 5.8 ± 3.4). Daytime sleepiness was on average only just within *normal* (ESS = 7.2 ± 3.9). Although not statistically significant (*F* (4, 211) = 1.5, *p* = 0.204), the highest scores were found among those *decided to act* (*M* = 8.4 ± 4.2) and the lowest scores among those *decided not to act* (*M* = 6.3 ± 3.3) and *acting* (*M* = 6.3 ± 3.7).

Mean DBAS score was 63.4 ± 22.6, with no statistically significant difference between the stages of the adoption process of sleep apps (*F* (4, 212) = 0.27, *p* = 0.900). Bedtime procrastination score was on average 2.94 ± 0.93 and did not differ significantly between the stages (*F* (4, 212) = 2.19, *p* = 0.072). However, regarding self-control (BSCS = 43.2 ± 8.0), a significant, but small effect was found for the stages of the adoption process of sleep apps (*F* (4, 212) = 2.47, *p* = 0.046, η^2^ = 0.05). Post hoc tests revealed a significant difference (*p* = 0.042) between those *unengaged* (*M* = 42.0 ± 7.5) and those *acting* (*M* = 46.5 ± 8.2) with a moderate effect (*d* = 0.58).

Results of the correlational analyses are depicted in Table 2. Age was negatively related to time in bed and total sleep time, while training volume showed positive correlations with sleep efficiency and self-control (BSCS) and negative correlations with poor sleep quality (PSQI) and bedtime procrastination (BPS). Despite significant correlations between sleep quality variables that constitute components of the PSQI (i.e. total sleep time, sleep efficiency), poor sleep quality was positively correlated with daytime sleepiness (ESS), sleep-related cognitions (DBAS), and bedtime procrastination (BPS), and negatively correlated with self-control (BSCS). Bedtime procrastination was further negatively correlated with sleep efficiency, positively with ESS, and negatively with BSCS. Furthermore, self-control showed positive correlations with sleep efficiency and ESS.

**Table 2:** Correlation patterns between sleep-related and demographic variables

		1	2	3	4	5	6	7	8	9
1	Age	–	–	–	–	–	–	–	–	–
2	TV	−0.03	–	–	–	–	–	–	–	–
3	TIB	−0.27^***^	0.06	–	–	–	–	–	–	–
4	TST	−0.27^***^	0.11	0.96^***^	–	–	–	–	–	–
5	SE	−0.08	0.19^**^	0.02	0.29^***^	–	–	–	–	–
6	PSQI	0.06	−0.16^*^	0.03	−0.14^*^	−0.58^***^	–	–	–	–
7	ESS	−0.04	−0.09	−0.05	−0.07	−0.07	0.36^***^	–	–	–
8	DBAS	−0.06	0.08	0.12	0.07	−0.14^*^	0.30^***^	0.18^**^	–	–
9	BPS	−0.06	−0.23^***^	−0.02	−0.09	−0.28^***^	0.34^***^	0.36^***^	0.04	–
10	BSCS	0.05	0.31^***^	−0.01	0.05	0.22^***^	−0.33^***^	−0.36^***^	−0.02	−0.63^***^

*TV* training volume (h/week), *TIB* Time in bed (min), *TST* Total sleep time (min), *SE* Sleep efficiency (TIB / TST × 100, in %), *PSQI* Pittsburgh Sleep Quality Index, *ESS* Epworth Sleepiness Scale, *DBAS* Dysfunctional Beliefs and Attitudes about Sleep Scale, *BPS* Bedtime Procrastination Scale, *BSCS* Brief Self-Control Scale

*p < 0.05, **p < 0.01, ***p < 0.001

## Discussion

The aim of the present study was to analyse the user behaviour of smartphone and wearable technologies in the context of recovery self-management and self-tracking among German athletes. The overall prevalence of sleep app users was with less than 20% surprisingly low. Considering different aspects such as sex, type of sport, competition participation, and training volume, no remarkable characteristics among users versus non-users were identified. In terms of the different types of apps, it seems that fitness apps were more popular than sleep apps followed by nutrition apps. The correlation between the sleep apps and the other two types of apps indicate that non-users of sleep apps are probably also non-users of fitness or nutrition apps. This applies also to the correlation between the use of sleep apps and wearable technologies. Comparing the survey results with those reported by König et al. (2018) for 1215 adults with a mean age of 41 ± 18 years and 64% female respondents, several differences can be observed regarding the adoption process of fitness apps and nutrition apps. While fitness apps were also more frequently used than nutrition apps in that study, only one quarter was identified as *acting* compared to almost one half of the current sample.

There were also no remarkable differences among sleep indices between sleep app users and non-users. However, self-control was found to be highest among sleep app users compared to non-users (i.e. stage 1: *unengaged*). As the application of self-tracking devices requires certain engagement by the users, higher self-control seems to support this behaviour. It may also be speculated that athletes with higher self-regulatory abilities are more willing to investigate further resources into their recovery self-management. The negative correlation between self-control and bedtime procrastination supports this assumption, as delaying bedtime becomes less likely with higher self-control ratings (Kroese et al., 2016). According to Choi et al. (2018), smartphone applications have the potential to raise awareness and promote healthy sleep habits and by this means may support sleep self-management. Unfortunately, the present survey does not provide further information about the type of sleep apps among users as well as those of the disengaged non-users and why they decided to disengage. Only few apps incorporate behavioural constructs to encourage healthy sleep hygiene (Grigsby-Toussant et al., 2017) and it seems worthwhile to examine the apps’ functionality and appeal to this group. Moreover, further investigation on the user’s motives would provide deeper insights. For instance, Roomkham, Lovell, Cheung, and Perrin (2018) identified five styles of personal tracking based on the users’ needs. Specifically, these (potentially overlapping) styles were classified as (a) directive tracking, (b) documentary tracking, (c) diagnostic tracking, (d) collection rewards tracking and (e) fetishized tracking. The concern that certain people may develop an unhealthy obsession with healthy sleeping that could be enforced by the risk of false-positive diagnoses by sleep apps raised by Van den Bulck (2015) apparently does not apply to the current athletic population. Thus, this phenomenon that Baron et al. (2017) described as “orthosomnia” probably is rather an issue for patients with diagnosed sleep disorders who indeed seek relief and optimisation of their sleep insufficiency. Thus, based on the current findings, consumer sleep tracking technology apparently cannot be considered a threat to sport science research and practice. On the contrary, it may comprise some potential which still has to be further explored. For instance, Reichert et al. (2021) discuss the potential of ambulatory assessment for precision psychiatry which can also be considered for applied sport science and sport psychological interventions. One advantage is that data can be assessed in real-time and real-life conditions over longer periods of time. However, users of commercial devices have to take possible lack of data security into account. On the other hand, large-scale data are available to evaluate physical activity and sleep behaviour on the population level. By this means, Rezaei and Grandner (2021) as well as Capodilupo and Miller (2021) were able to examine trends in sleep and physical activity before and during COVID-19 pandemic developments retrospectively by using consumer wearable technology data from Fitbit (Fitbit Inc., San Francisco, CA, USA) and WHOOP (WHOOP, Boston, MA, USA), respectively. Nevertheless, most of the available commercial activity and sleep technologies lack sufficient data on reliability and validity in terms of detecting sleep parameters (Khosla et al., 2018), why it is recommended to rely on empirically evaluated devices (Halson, 2019). There is quite a wide range of ambulatory assessment methods for sleep monitoring (in athletes) so that the most convenient approach can be chosen according to the purpose. Portable polysomnography (Hof zum Berge et al., 2020) and actigraphy (Sadeh, 2011) evolved as promising methods to investigate sleep behaviour in an ecologically valid way.

Regarding the sleep quality of the participants, the average PSQI score was just around the cut-off (*M* = 5.3), with the majority categorised as *good* sleepers (59%). The score was higher than that of a German sample of adults below 40 years (*M* = 4.04 ± 2.73, *n* = 509), and prevalence of *good* sleep quality was lower than that of a general community sample (64.1%) according to Hinz et al. (2017). However, comparing the present findings to another sub-elite athletic population (*n* = 146), prevalence of *good *sleep quality was higher than the 35% reported by Doherty et al. (2021). It needs to be acknowledged, though, that Doherty et al. (2021) set the cut-off at PSQI ≥ 5, while the current study applied the more conservative cut-off with PSQI ≥ 6 (Buysse et al., 1989; Hinz et al., 2017). Comparing the findings to those of Bender, Van Dongen, and Samuels (2019), similar PSQI scores for the athletic sample (*n* = 63) were found (*M* = 5.0 ± 2.6).

Taking also the findings of the reported habitual sleep duration (*M* > 7 h) and the normal amount of daytime sleepiness (79% with ESS ≤ 10) into account, it may be assumed that the current sample was not chronically sleep deprived. It should be considered that the survey was conducted in a timeframe of social- and sport-restricted regulations during the 2020 COVID-19 pandemic activities. As university classes took place online and most of the employees were working at home, individuals were more flexible in terms of scheduling their leisure activities and sleep behaviour. This may have enhanced sleep quality and/or quantity, as Blume, Schmidt, and Cajochen (2020) reported positive effects on sleep–wake patterns in European adults between 26 and 35 years. In addition, Wright Jr. et al. (2020) found increased sleep regularity and sleep quantity among US American university students during COVID-19 lockdown orders. Furthermore, an Australian survey among elite and sub-elite athletes during the lockdown period support the current findings, as the athletes reported spending more time in bed and sleeping longer than before the lockdown (Facer-Childs et al., 2021). Future research should further explore the impact of the pandemic and its consecutive regulations on physical activity patterns, mental health, quality of life and sleep in elite athletes. A systematic review revealed that overall physical fitness and training volume (i.e. number of days, duration) as well as sleep quality decreased, while negative emotions (stress, fatigue, depression) increased (Jurecka, Skucińska, & Gądek, 2021).

Moreover, findings on bedtime procrastination were comparable to scores of Dutch adults (*M* = 2.7 ± 0.8) and even slightly lower than those of a Polish sample (*M* = 3.2 ± 0.9) according to Kroese et al. (2016) and Herzog-Krzywoszanska and Krzywoszanski (2019), respectively. Unfortunately, there is currently no cut-off to identify the problematic degree of postponing going to bed and a lack of comparable athletic samples that may help interpret the present findings. It may be generally assumed that getting insufficient sleep is a typical problem in athletes and that sleep is lacking sufficient prioritisation (Halson & Lastella, 2017).

Considering sleep-related cognitions, the average DBAS score (*M* = 64.4) was slightly higher compared to the baseline scores of two groups in a study with physically active university students (group 1: *M* = 52.4 ± 18.1, *n* = 25, group 2: *M* = 56.4 ± 26.6, *n* = 33) reported by Kölling and Hof zum Berge (2020). Moreover, the score of the present study corresponds to the cut-off that distinguishes between *normal* and an *unhelpful* degree of sleep-related beliefs (Carney et al., 2010). Interestingly, only those categorised as users of sleep apps (i.e. *acting*) were identified with a *normal* DBAS score. Nevertheless, the difference between the groups was marginal so that mean values should be considered cautiously. While the effect was small, dysfunctional beliefs were correlated with poor sleep quality and higher daytime sleepiness.

Several limitations need to be addressed. The participants were recruited via a convenient sample and the rather small number does not allow for generalisations. Moreover, the self-ratings may be biased by subjective recall deficiencies, on the one hand, and they constitute only a snapshot, on the other hand. Longitudinal analyses of sleep and recovery behaviour before, during and following lockdown restrictions would provide more detailed insights. The effect of COVID-19 regulations was not the focus of this study, but the results need to be interpreted with the current situation in mind. Follow-up studies that examine recovery self-management activities and sleep patterns during subsequent lockdowns and ‘new normal’ situations will help understand the interplay between recovery and external influencing factors to derive better guidance for athletes and practitioners.

## Conclusion

Overall, the results of this study provide interesting insights and allow encouraging conclusions. On the one hand, the present sample seems to be rather unaffected by smartphone and wearable self-tracking technologies so that most of them do not take the risk of the potentially unsubstantiated or even dangerous feedback on their sleep (Ko et al., 2015). This implies that these athletes are not as vulnerable as expected and this may enhance their compliance in the collaboration with sport scientific researchers who are using empirically validated devices. Participants’ attitudes toward sleep monitoring and intervention approaches may probably not be affected by non-validated dubious feedback and dysfunctional self-treatment approaches. Thus, researchers and practitioners will probably not be confronted with contradicting and conflicting occasions regarding monitoring approaches and data interpretation. On the other hand, problematic sleep seems to be an issue for the minority of the sample. Nevertheless, those affected athletes should raise concern as sleep is an essential factor contributing to performance as well as recovery (Walsh et al., 2021). Therefore, sleep monitoring may be a possible approach to raise the athlete’s awareness and to detect dysfunctional sleep indices at an early stage (Halson, 2019). While a perfect sleep assessment method does not exist, several methods should be combined to balance their advantages and disadvantages (Ibáñez, Silva, & Cauli, 2018). Moreover, Halson (2019) highlights that practitioners and athletes should be aware of the limitations of different monitoring methods, especially smartphone applications and consumer-based technologies.

## Declarations

For this article no studies with human participants or animals were performed by any of the authors. All studies mentioned were in accordance with the ethical standards indicated in each case.
